# Combined Target-Immobilized and Library-Immobilized SELEX for Selecting High-Affinity α-Amanitin Aptamers

**DOI:** 10.3390/toxins18040163

**Published:** 2026-03-30

**Authors:** Yang Li, Muling Shi, Wenyue Li, Yiqing Yang, Xiang Li, Chen Shen, Xiong Wang, Shuanglin Zhang, Jie Du

**Affiliations:** 1 State Key Laboratory of Tropic Ocean Engineering Materials and Materials Evaluation, School of Materials Science and Engineering, Hainan University, Haikou 570228, China; 23210805000002@hainanu.edu.cn (Y.L.); wenyueli@hainanu.edu.cn (W.L.); dujie@hainanu.edu.cn (J.D.); 2 Molecular Science and Biomedicine Laboratory, College of Biology, Hunan University, Changsha 410082, China; 3 Hunan Provincial Key Laboratory of Forestry Biotechnology, College of Life Science and Technology, Central South University of Forestry & Technology, Changsha 410004, China; yyq970504@163.com (Y.Y.); lx534976886@gmail.com (X.L.); charybdis233@gmail.com (C.S.); wangxiongvincent@163.com (X.W.); zslzlt@163.com (S.Z.)

**Keywords:** amanita toxins detection, α-amanitin, small molecules, aptamers, target-immobilized SELEX, library-immobilized SELEX, human serum albumin

## Abstract

Deaths from the accidental ingestion of poisonous *Amanita* mushrooms occur every year due to the lack of a specific antidote against α-amanitin poisoning. Intervention and treatment can be promptly carried out to avoid serious consequences when the toxin can be effectively detected in whole blood before liver toxicity develops. Aptamers are molecular recognition units similar to antibodies, capable of specifically recognizing and detecting small molecules such as α-amanitin for which monoclonal antibodies are difficult to prepare. However, α-amanitin has a small molecular size and limited binding sites, which bring difficulties to aptamer selection. Moreover, achieving highly specific detection of α-amanitin in whole blood remains challenging due to the presence of potentially interfering components, such as human serum albumin (HSA). For these problems, we propose an aptamer selection method for small-molecule target α-amanitin, combining target-immobilized and library-immobilized SELEX to select high-affinity aptamers. To exclude HSA interference, counter-selection was introduced to remove HSA-bound sequences. Through these strategies, we successfully selected a highly specific α-amanitin aptamer with nanomolar affinity.

## 1. Introduction

α-Amanitin is the most toxic toxin in *Amanita phalloides*, with a lethal dose to humans being around 0.1 mg/kg [1], its chemical structure is shown in Figure S1 [2]. α-Amanitin is rapidly absorbed from the gastrointestinal tract into the blood after poisoning [3]. It can then be transported to organs like the liver and kidneys, causing hepatic and renal damage [1]. Early blood detection of α-amanitin enables timely intervention. Thus, developing a highly specific technique for detecting α-amanitin in blood is crucial for early diagnosis.

The detection techniques currently used for the plasma or serum of α-amanitin mainly include liquid chromatography-tandem mass spectrometry (LC-MS/MS), molecularly imprinted sensors, immunosensors, and enzyme-linked immunosorbent assays (ELISA) [4,5,6,7]. LC-MS/MS offers high sensitivity and specificity for individual analytes, but this method requires trained personnel and uses expensive instruments. Molecularly imprinted sensors have exhibited high selectivity for α-amanitin. Yet template leakage risks affect detection accuracy. Immunosensors using monoclonal antibodies against α-amanitin, which can specifically detect both α-amanitin and β-amanitin simultaneously. Nevertheless, the preparation of such monoclonal antibodies is technically challenging, costly, and time-consuming. Additionally, a commercial ELISA kit that is available year-round enables the detection of α-amanitin and γ-amanitin down to 0.2 ng/mL, demonstrating high selectivity. ELISA does not require expensive laboratory equipment; however, it remains limited in preparing monoclonal antibodies against small molecules such as α-amanitin. Despite progress in α-amanitin blood detection, a method that is easy to operate, prepare, low-cost, portable, and highly sensitive and specific is still lacking. Fortunately, aptamers offer a new opportunity for developing α-amanitin detection technology.

Aptamers have a lot of special advantages, including facile chemical synthesis and modifications, high stability and specificity, cost-effective production, and adaptability to test strips [8]. They are widely applied in biochemical analysis, specifically in toxin detection [9,10,11]. Notably, aptamers require a thermal denaturation step to eliminate non-specific secondary structures. This step relies on heating appliances and adds an incubation step. Optimizing the aptamer sequence reduces its dependence on thermal folding and further enhances its applicability for portable instruments. High-affinity aptamer selection is crucial for specific detection technologies. However, selecting aptamers for small molecules like α-amanitin (918.97 Da) is harder than for large molecules such as proteins [12,13,14]. Their small size significant reduces molecular mass differences between aptamer-target complexes and unbound sequences; their simple structures offer fewer binding sites and weak aptamer interactions, complicating complex separation [12,14]. Thus, small-molecule aptamer selection typically requires immobilizing the target on solid substrates, like beads [15], microplate wells [16], and columns [17]. For example, Strzałka et al. covalently immobilized modified α-amanitin on cyanogen bromide-activated Sepharose 4B, then selected aptamers with a dissociation constant (K_d_) of 5.026 ± 0.69 μM [18]. Yet small molecule targets often lack functional groups for coupling to solid surfaces, even feasible immobilization may alter the target’s natural structure or block critical binding sites due to chemical modifications during immobilization, reducing affinity [19].

An alternative small-molecule aptamer selection strategy is library-immobilized SELEX, which avoids small-molecule conjugation by immobilizing the library onto the substrate via complementary base pairing. For instance, Li et al. proposed a novel library-immobilized SELEX using positively charged gold nanoparticles to select an α-amanitin aptamer [20]. This method has successfully selected aptamers for small molecules like polymyxin B and spermidine [21,22]. Nevertheless, this approach suffers from low enrichment. Immobilized libraries may undergo dynamic dissociation due to weak internal base pairing, causing some unbound sequences to detach from the support and leading to false positives. Additionally, library abundance may decrease, and diversity cannot be guaranteed during selection [23]. In short, both strategies have limitations when used alone for selecting aptamers with affinity for small molecules.

In the study, our goal was to develop a strategy combining target-immobilized and library-immobilized SELEX for aptamer selection, aiming to maximize library diversity and stability via target-immobilized SELEX while minimizing the impact of the target’s natural conformational changes on selection results through library-immobilized SELEX. Initially, retain maximal abundance of enriched sequences using target-immobilized SELEX, then combine affinity candidate sequences with a new random library to form a combinatorial library, and finally conduct library-immobilized SELEX for further selection to obtain aptamers with strong affinity and high specificity.

Aptamer-based specific detection technologies require not only high-affinity aptamers but also overcoming interference in complex biological matrices in practical detection. Current α-amanitin detection methods are mainly applied to mushroom or spiked urine samples [24,25]. In 2022, Li et al. developed an electrochemical sensor with gold-graphene quantum dot nanohybrid and DNA cyclic dual-signal amplification, enabling the detection of spiked α-amanitin isolated from blood samples [26]. Nevertheless, highly specific α-amanitin detection in blood remains challenging due to HSA, which accounts for approximately 60% of total plasma proteins [27]. With multiple binding sites and strong adsorption, HSA causes non-specific aptamer binding, compromising detection accuracy. Thus, in this study, HSA-modified tosyl-activated magnetic beads were used for counter-selection to reduce non-specific binding from HSA.

**Figure 1:**
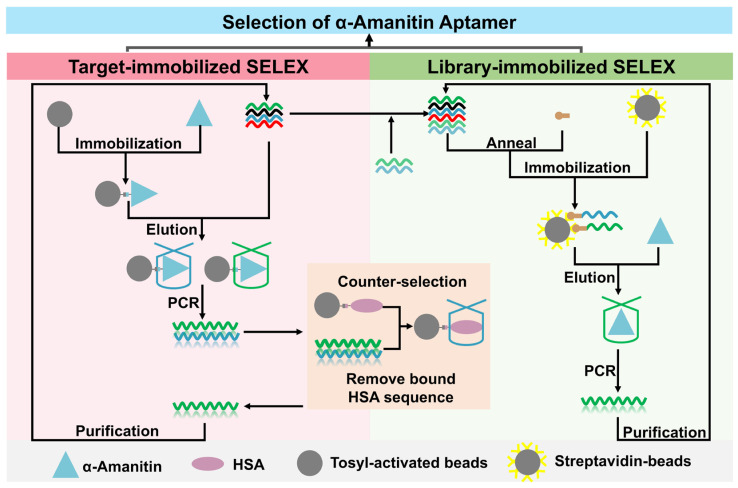
Schematic of α-amanitin aptamer selection via target-immobilized SELEX and library-immobilized SELEX.

The α-amanitin aptamer selection process is shown in Figure 1. α-amanitin was immobilized onto tosyl-activated magnetic beads. The random library was mixed with the α-amanitin-bead solution, and ssDNA bound to the target was obtained by magnetic separation and amplified for multiple rounds. From the 4th round, counter selection was conducted every two rounds to remove sequences bound to HSA. After 14 rounds, high-throughput sequencing (HTS) was performed on enriched ssDNA libraries from rounds eight and fourteen of target-immobilized SELEX, identifying high-affinity aptamer candidates. Then, the initial ssDNA library selected from target-immobilized SELEX was mixed with new random sequences to form a new combinatorial library; hybrids of this ssDNA combinatorial library and biotin-modified oligonucleotides were incubated with streptavidin magnetic beads for library immobilization. α-amanitin solution was then added to incubate with the library, forming the librarytarget. The librarytarget was subsequently amplified and prepared as ssDNA to obtain a secondary library. After five rounds, we analyzed homology, secondary structures, and preliminarily evaluated affinities of the aptamer candidates. Finally, we used HSA as a counter-selection target to evaluate the specificity of aptamer, since HSA is the most abundant protein in human serum.

## 2. Results and Discussion

### 2.1. Selection of α-Amanitin Aptamers Through the Target-Immobilized SELEX

#### 2.1.1. Immobilization of Target α-Amanitin

**Figure 2:**
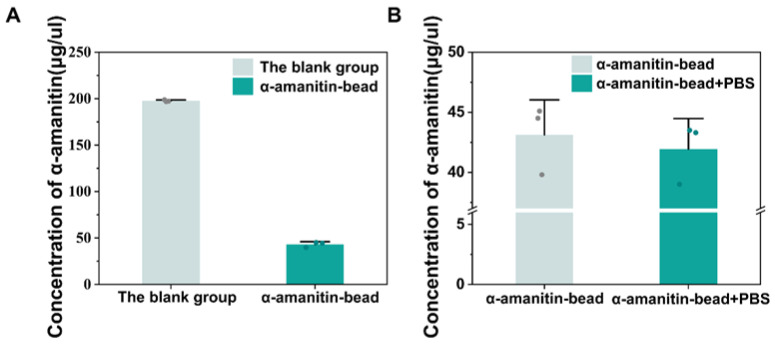
Evaluation of the immobilization level of α-amanitin onto tosyl-activated magnetic beads. (**A**) Concentration of α-amanitin in the supernatant before and after incubation with the beads; (**B**) concentration of α-amanitin in the supernatant before and after washing the α-amanitin-beads with PBS. Each experiment was repeated at least three times, error bars in this figure represent SD, and circles represent individual data points.

In the target-immobilized SELEX process, as α-amanitin itself has an active group that can be covalently coupled with a tosyl group, it was incubated with tosyl-activated magnetic beads overnight under controlled conditions (37 °C, pH 8.5) to form an α-amanitin-bead solution; meanwhile, α-amanitin without magnetic beads was used as the blank control. The α-amanitin concentrations in the supernatant before and after incubation directly indicated the α-amanitin bead immobilization efficiency. After incubation, if α-amanitin is successfully fixed on the tosyl-activated magnetic beads, the amount of α-amanitin in the solution will decrease. Therefore, the concentration of α-amanitin before and after incubation was measured using a Nanodrop 2000 spectrophotometer (Thermo Fisher Scientific, Waltham, MA, USA). As shown in Figure 2A, an average of 78.2% of the α-amanitin was bound to the beads compared to the blank control group, which indicated that the tosyl-activated magnetic beads had a high immobilization efficiency for α-amanitin. Then, the α-amanitin beads were washed with PBS buffer. The concentrations of the supernatant were measured before and after washing to calculate the percentage of α-amanitin eluted. As shown in Figure 2B, only 2.78% of α-amanitin was eluted from the beads, indicating that α-amanitin was firmly immobilized to them, and from that it could be demonstrated that a higher level of α-amanitin immobilization was achieved on the tosyl-activated magnetic beads.

#### 2.1.2. In Vitro Selection of Aptamers for α-Amanitin Through the Target-Immobilized SELEX

As a small molecule, the process of immobilizing α-amanitin onto beads easily induces non-specific binding of aptamers. α-amanitin has a limited number of binding sites, which contributes to low binding affinity between aptamers and the target. These two factors together make the selection of α-amanitin-specific aptamers challenging. In this study, the Oligo software package, version 7 (National Biosciences Inc., Cascade, CO, USA) was utilized to design library sequences. The ssDNA sequence library was 78 bases in length, with 40-nucleotide-length random sequences flanked by constant regions, providing a diversity capacity of 10^13^–10^15^. In addition, the primers rich in G/C bases and with high annealing temperatures were used in the primer design in order to minimize primer dimer formation and non-specific amplification. Maintaining strict conditions during aptamer selection is crucial for obtaining high-affinity aptamers [28]. Here, a total of 14 rounds of selection were conducted to obtain aptamers with high affinity and specificity, during which more stringent selection conditions were progressively implemented as the number of rounds increased (Table 1): increasing the elution times, allowing free sequences without affinity to be washed away; the decreasing amounts and shortening incubation time of ssDNA and α-amanitin reduced the probability of binding between non-specificity ssDNA and α-amanitin.

Furthermore, in the early stages of α-amanitin poisoning, α-amanitin rapidly enters the blood circulation and exists in human plasma [29]. However, human serum albumin (HSA), which is abundant in plasma, nonspecifically interacts with various endogenous or exogenous ligands or detection probes [30], thereby interfering with the specific recognition of α-amanitin. Aptamer selection can be modified by introducing counter selection, which not only helps eliminate sequences that non-specifically bind to HSA, but also increases the stringency of aptamer-target binding conditions. Thus, since the completion of the 4th round of selection, a conditional control group of HSA-modified tosyl-activated magnetic beads was used for counter selection in every two rounds to remove nonspecific sequences. These aforementioned strategies all contributed to selecting high-specificity and high-affinity aptamers for target-immobilized SELEX.

**Figure 3:**
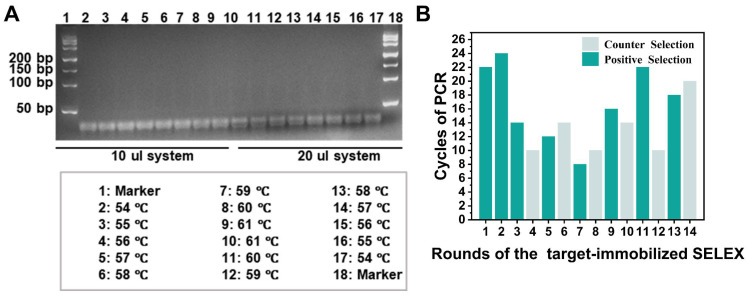
Optimization of PCR conditions in target-immobilized SELEX. (**A**) Optimization of PCR reaction volume and annealing temperature; (**B**) optimization of the number of PCR cycles.

To ensure the accuracy of the ssDNA selected in each round, the PCR products of each round of selection were verified by agarose gel electrophoresis. The reaction volume, annealing temperature, and the number of PCR cycles were optimized during the amplification step (Figure 3). In addition, the bands in the 10 μL system were clearer than those in the 20 μL system. The results show that the bands of aptamer sequences were all the clearest and without smearing at 57 °C, compared with other temperatures (54 °C, 55 °C, 56 °C, 58 °C, 59 °C, 60 °C, and 61 °C) (Figure 3A). Regarding the number of PCR cycles, in the positive selection, the efficient selection process was also reflected by the decreasing number of PCR cycles required for the positive selection. At the same time, after each round of counter selection, the number of PCR cycles required for the amplification of bound DNA using the counter selection beads increased. These results suggested that the high specificity and affinity of aptamers led to a growing number of aptamer candidates specifically bound to α-amanitin by the end of each selection round. These results suggested that the high-specificity and affinity aptamers were specifically bound to α-amanitin by the end of the selection (Figure 3B).

**Figure 4:**
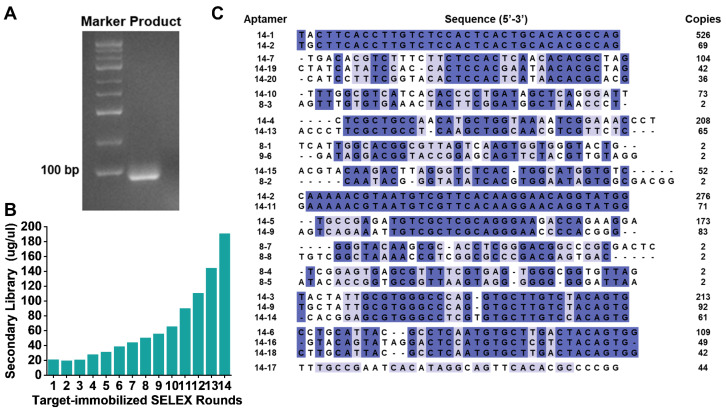
(**A**) Identification of PCR products after target-immobilized SELEX; (**B**) secondary library concentration of α-amanitin-bound ssDNA after each target-immobilized SELEX selection round; (**C**) homology analysis of α-amanitin candidate aptamer sequences selected in 8 and 14 rounds during the target-immobilized SELEX. One-hundred percent homologous bases are labeled with a dark blue background, and 50% homologous bases are labeled with a light blue background. Hyphen (-) represents a gap in the sequence alignment, indicating a position where no nucleotide base is present in the corresponding aptamer sequence.

Finally, a random sample was taken to verify the amplification. Figure 4A showed that the product bands were located at 75 bp, with single and bright bands, without non-specific bands and dispersion. Additionally, as depicted in Figure 4B, secondary library concentration positively correlates with the selection rounds. The secondary library showed a specific and progressive enrichment from round 1 to round 14. Notably, after the 14th selection round, the concentration of the secondary library substantially exceeded that of the preceding 13 rounds, reaching 190.7 μg/μL. Moreover, after 14 rounds of selection, the concentration of the secondary library no longer increased. Therefore, the selection process was terminated at the 14th round.

### 2.2. High-Throughput Sequencing and Sequence Analysis of the Enriched Library

To identify aptamers enriched through the target-immobilized SELEX, high-throughput sequencing was carried out on the PCR products obtained from the initial screening stage (the eighth round) and the later stage (the fourteenth round). The sequencing results showed that in the 8th round, 124,892 distinct sequences were detected in total, among which only eight sequences were repeated, and each was repeated only twice. In contrast, a total of 116,489 different sequences were detected in the 14th round, 528 sequences were repeated multiple times, and their repetition frequency was much higher than that in the 8th round. At the same time, the sequencing results were analyzed for quality. Taking Seq14-2 as an example (Figure S2), FastQC results showed that the sequence had a high-quality score, as most of the bases were scored above 30. The quality of the Illumina sequencing data is highest for the first 150 bp, while most of the sequences were located within the 25~60th positions in the sequencing reads. Subsequently, for homology analysis, we selected all repetitive sequences from the 8th round and the top 20 repetitions in the 14th round (ssDNA library: 5′-GGGAGCTCAGAATAAACGCTCAA-35N-TTCGACAT-3′) and then constructed a phylogenetic tree using MEGA X software (https://www.megasoftware.net/, accessed on 4 March 2025). A cluster analysis of sequence similarity was performed, and the sequences were grouped based on their nucleotide sequences, as shown in Figure 4C. Sequences in the 14th round exhibited high similarity; however, the similarity between sequences from the 8th round was relatively low, implying that sequences with high repetition frequencies were increasingly selectively retained and amplified during the screening process. These results, to some extent, confirmed that the target-immobilized SELEX could effectively enrich specific aptamer sequences.

**Figure 5:**
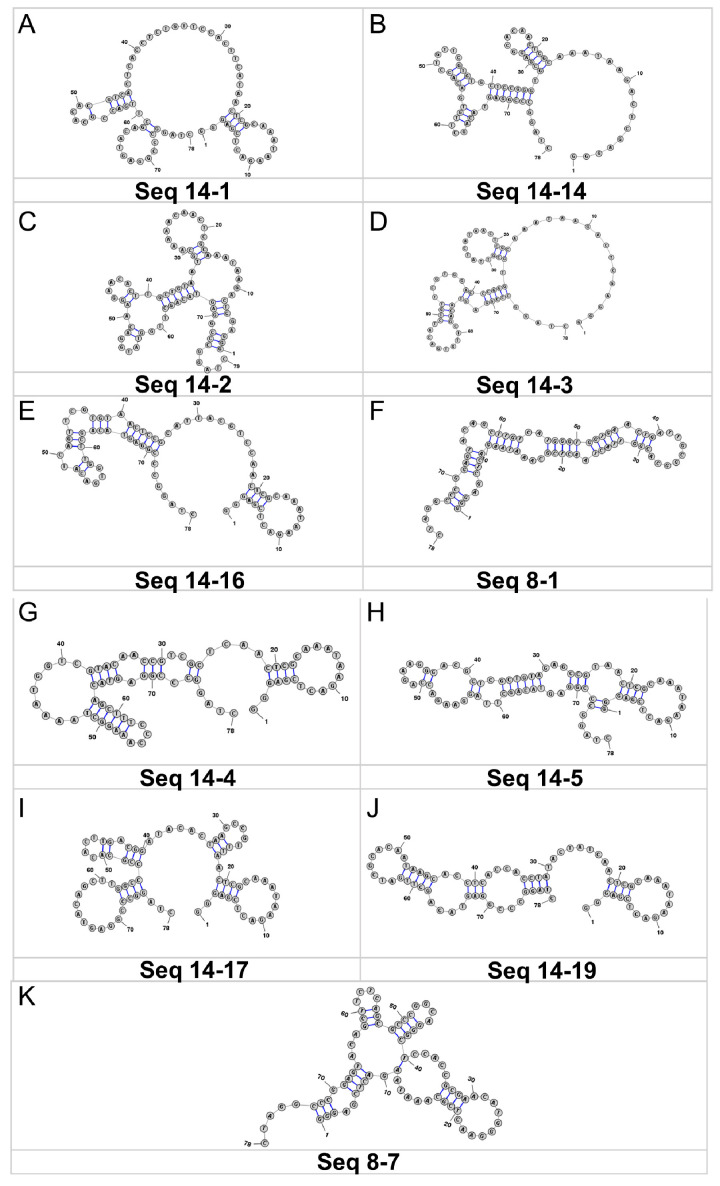
Secondary structures of candidate aptamers with sequence validity potential ranking. Structures (**A**–**K**) are ordered by decreasing validity potential, representing aptamers (Seq14-1, Seq14-14, Seq14-2, Seq14-3, Seq14-16, Seq8-1, Seq14-4, Seq14-5, Seq14-17, Seq14-19, and Seq8-7).

Aptamers are able to specifically recognize specific targets, a property that relies on their special secondary structure. Therefore, the analysis of the secondary structure of aptamers facilitates rapid and accurate selection of α-amanitin aptamers with high affinity. Based on the results of the target-immobilized SELEX, mFold software (http://mfold.rna.albany.edu/?q=mfold/DNA-Folding-Form, accessed on 4 March 2025) was used to predict the secondary structure of part of the highly conserved sequences selected in rounds 8 and 14 (Seq8-1, Seq8-4, and Seq8-17 in Round 8; Seq14-1, Seq14-2, Seq14-3, Seq14-4, Seq14-5, Seq14-6, Seq14-7, Seq14-10, Seq14-14, Seq14-15, Seq14-16, Seq14-17, andSeq14-19 in Round 14); as well as a complete sequence containing two fixed sequences). According to the simulation results of the secondary structure presented in Tables S1 and S2, the secondary structure simulated by random sequences and complete sequences with fixed sequences can be divided into eight categories. These sequences were found to have relatively abundant stem-loop structures, which provide sufficient sites for the specific binding of aptamer and α-amanitin. Above all, we selected the sequences with the largest abundance of secondary structures (Seq8-1, Seq14-1, Seq14-2, Seq14-4, Seq14-5, Seq14-6, Seq14-14, Seq14-17, Seq14-19, Seq14-3, and Seq8-7) as random libraries for the library-immobilized SELEX, and ranked them by sequence validity (Figure 5).

#### Binding Measurement of Enrichment Sequences

**Figure 6:**
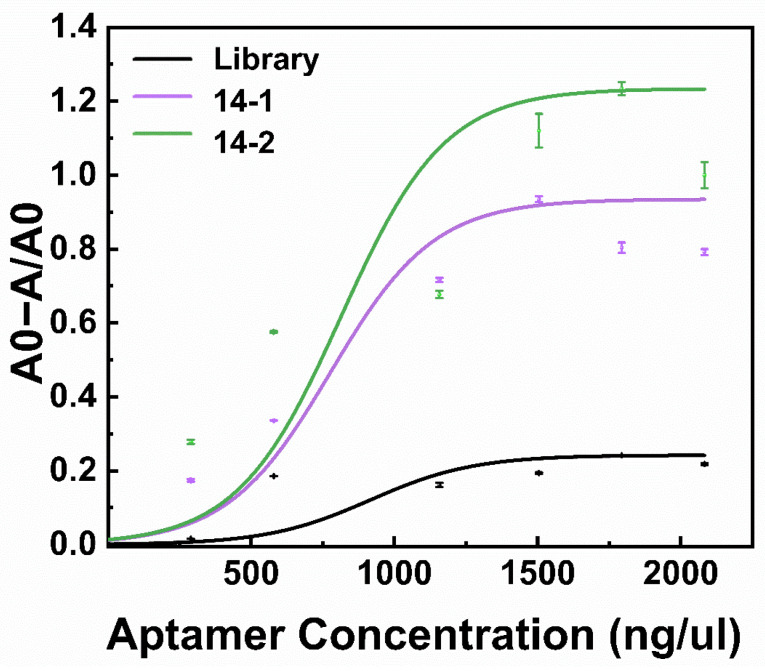
Characterization of the affinity of aptamer 14-1 and aptamer 14-2 for α-amanitin. A0 and A represent the input aptamer concentration and the concentration of aptamers remaining in the supernatants after incubation with α-amanitin-beads, respectively. The purple and green lines represent the standard curve of aptamers 14-1 and 14-2 with α-amanitin, respectively. The black line represents the standard curve of a random library with α-amanitin.

The binding affinities of two randomly selected aptamers were analyzed. Aptamers and the library were incubated with different concentrations of α-amanitin beads for 30 min at room temperature. Next, the binding rate was calculated on the basis of the difference between the input aptamer concentration used in the experiment and the concentration of aptamers that remained in the supernatants after incubation with the α-amanitin beads. The binding rate of the aptamer was enhanced as the target concentration increased, as shown in Figure 6. The dissociation constant (K_d_) values were 1.88 mM and 1.85 mM for Seq14-1 and Seq14-2, respectively. The results showed that both aptamers exhibited higher binding affinities than those of the initial library sequence, indicating that higher-affinity aptamers were initially successfully selected by the target-immobilized SELEX.

### 2.3. Selection of α-Amanitin Aptamers Through the Library-Immobilized SELEX

#### Immobilization of ssDNA Library

**Figure 7:**
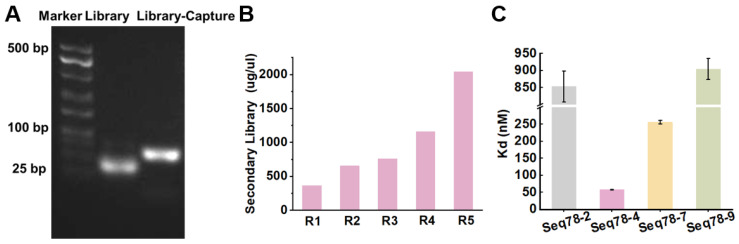
(**A**) Identification of PCR products before and after hybridization of the library and capture sequences in the library-immobilized SELEX; (**B**) secondary library concentration of α-amanitin-bound ssDNA after each library-immobilized SELEX selection round; (**C**) affinity evaluation of candidate aptamers for α-amanitin. Sequences selected in 8 and 14 rounds during the target-immobilized SELEX.

During the library-immobilized SELEX process, when the ssDNA library was being immobilized, the target α-amanitin remained free. To achieve optimal library immobilization, the library must hybridize effectively with the capture sequence, which is essential for maximizing immobilization efficiency. Libraries were mixed with capture at a ratio of 1:1.2, followed by thermal denaturation and renaturation. A 3% agarose gel electrophoresis was employed to assess the hybridization of the library and capture sequences. As illustrated in Figure 7A, on the agarose gel electrophoresis, the library exhibited a bright and wide band (lane 2) at approximately the 50 bp size location. This characteristic band pattern indicates a high level of sequence diversity within the library, making the library suitable for the subsequent selection process. The band in the lane corresponding to the library-capture sequence mixture on the agarose gel had a larger molecular weight compared to that of the ssDNA library, which suggests that the ssDNA successfully hybridized with the capture sequences.

After the ssDNA library was immobilized on the beads, the library beads were washed four times per round before incubation with α-amanitin to improve overall selection efficiency. The unbound ssDNA in the supernatant was measured after each wash. As shown in Figure S3, the concentration of unbound ssDNA in the supernatant nearly approached zero after four washes, which effectively reduced the retention of non-binding sequences and enhanced the selection efficiency.

As α-amanitin was introduced into the immobilized ssDNA library, the bound ssDNA folded into a three-dimensional conformation, which caused the bound ssDNA to be released from the beads. After magnetic separation, the released ssDNA was obtained and subsequently amplified for the next round of selection. By repeating this selection procedure, aptamers with high affinity and selectivity could be enriched. To enhance the affinity and specificity of aptamer selection, we accordingly adjusted the experimental conditions as the selection rounds progressed. On the one hand, we introduced counter SELEX to apply more pressure for the selection of highly-specific ssDNA sequences. On the other hand, we optimized the number of PCR amplification cycles for each round to minimize the amplification of non-specific products. In Figure S4, the required number of PCR cycles significantly decreased as the number of selection rounds increased. For instance, only 14 PCR cycles were required to obtain sufficient copies for follow-up selection. This suggested that specific aptamers were enriched as the SELEX rounds progressed.

Eventually, to further evaluate the enrichment of α-amanitin aptamers, secondary libraries from each round were collected, and their concentrations were measured. As depicted in Figure 7B, the concentrations of the secondary libraries increased with the selection rounds. The 5th secondary library concentration was the highest among all rounds, and increased five-fold compared to the first round, reaching 2041.6 μg/μL. This result indicated that the aptamers were successfully enriched in the 5th round pool.

### 2.4. High-Throughput Sequencing and Sequence Analysis of Candidate Aptamers

We performed high-throughput sequencing on the PCR products obtained from the last round of selection. The sequencing results showed that a total of 1.44 × 10^5^ sequences were obtained. Among these sequences, 1.1 × 10^5^ sequences with lengths of 78 nt or more were identified, and the number of repeated sequences among these 1.1 × 10^5^ sequences accounted for approximately 15.1%. Nine sequences with the highest repetition frequencies were selected. Table S3 shows the sequences with the fixed primer regions removed, designated as Seq78-1 to Seq78-9.

**Figure 8:**
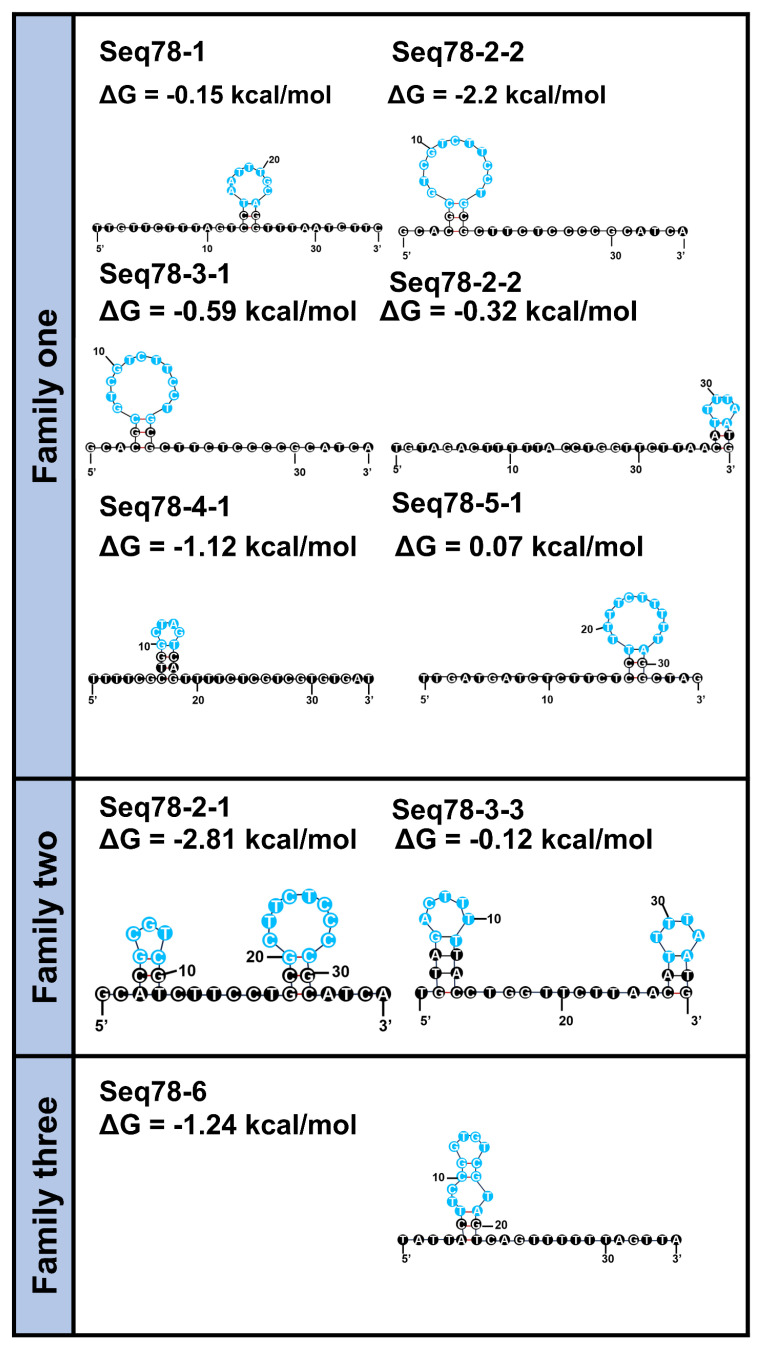
Three families of predicted partial secondary structures of the nine most frequently repeated aptamers in the library-immobilized SELEX. The diagram shows the predicted secondary structure of each aptamer by mFold software, with blue circles representing loop regions. The corresponding free energy change (ΔG, in kcal/mol) for each structure is presented below the sequence, indicating the thermodynamic stability of the secondary structure.

Subsequently, the secondary structures of these nine sequences were predicted using mFold software with default parameters. These structures were grouped into three families according to their structural similarities. The predicted secondary structures for all candidates, including their Gibbs free energy (ΔG) values, are provided in Figure 8 and Figure S5. The secondary structure of each group of aptamers revealed a typical stem-loop structure and lower ΔG. The stem-loop structure will promote the recognition and binding of aptamers with small molecule α-amanitin [31]. In addition, the lower the ∆G, the more easily the sequences could fold into the predicted structure. Thus, we selected one or two representative sequences with low ΔG from each family as candidate sequences for affinity determination.

### 2.5. Investigation of Binding Affinity and Specificity

The binding affinity between an aptamer and its target is expressed by the equilibrium dissociation constant (K_d_) [32]. To evaluate the binding affinity of the above aptamers, seven candidate aptamers were immobilized on beads to form library beads; subsequently, α-amanitin was incubated with the library beads, and the supernatant was collected after magnetic separation. Initial concentrations of the seven candidate aptamers and α-amanitin, as well as the concentration of the library target in the supernatant, were measured, and the K_d_ values were calculated. Figure 7C shows that candidate aptamers (Seq78-2, Seq78-4, Seq78-7, and Seq78-9) have good affinity for α-amanitin, with K_d_ values of 850 ± 45 nM, 58 ± 1 nM, 256 ± 5 nM, and 910 ± 31 nM, respectively. Table S4 shows that these aptamers have affinities comparable to those of other α-amanitin [18,24,25,33]

In addition, the gold nanoparticle (AuNP)-based method is an effective method for testing aptamer K_d_ [24]. The affinity constants of the four sequences were also analyzed (Figure S6). As the aptamer concentration increased, protection of the AuNPs by the aptamer was enhanced. When the AuNPs encountered NaCl, the AuNPs maintained the original red color, and the higher the affinity, the more obvious the colloidal gold coagulation. Aptamer affinity was analyzed using GraphPad Prism 8 software, and the K_d_ values of Seq78-2, Seq78-4, Seq78-7, and Seq78-9 were 820 ± 5 nM, 60 ± 3 nM, 216 ± 3 nM, and 881 ± 6 nM, respectively. Although there are slight differences in experimental details between our study and the standard AuNP-based methods, these variations have a negligible influence on the reliability of K_d_ determination and do not alter the binding affinity trend of these aptamers. This method is especially suitable for the preliminary rapid screening of candidate aptamers in the early stage of SELEX screening, and its rationality and feasibility have been verified by relevant published studies [34,35]. It can reliably reflect the relative binding ability of aptamers and targets and fully meet the experimental requirements of this study for preliminary aptamer screening.

**Figure 9:**
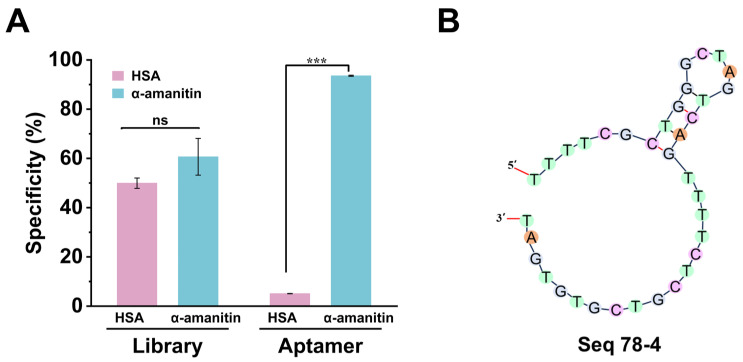
(**A**) Specificity evaluation of aptamer Seq78-4 for α-amanitin. (ns indicates non-significant differences, *** indicates *p* = 0.001); (**B**) The predicted secondary structures of aptamer Seq78-4.

The keys to achieving specific detection of α-amanitin lie in the aptamer’s high affinity toward α-amanitin and its ability to reduce non-specific binding to the HSA. Thus, we investigated the specificity of Seq78-4, the aptamer with the highest binding affinity, for α-amanitin, setting HSA as a negative control. We incubated α-amanitin and HSA separately with the library beads, followed by magnetic separation to collect the supernatants. Meanwhile, we measured the concentration of the aptamer before incubation (C0) and after incubation (C) with the targets, and the specificity was evaluated by the ratio C/C0. The specific analysis results are shown in Figure 9A. The concentration of the aptamer Seq78-4 binding with α-amanitin exceeded that measured in the HSA, which indicated that there was no cross reaction with the HSA and that the aptamer had high specificity (93.54%). The results validated that the aptamer Seq78-4 could effectively exclude the interference of HSA. Therefore, sequence 78-4 was identified as an effective α-amanitin aptamer, with the following nucleotide sequence: 5′-TTTTCGCTGGCTAGTCAGTTTTCTCGTCGTGTGAT-3′. It folds into a short stem-loop secondary structure formed by a small number of Watson–Crick base pairs, with a calculated ΔG value in the negative range that endows the aptamer with moderate structural stability and proper conformational flexibility.

### 2.6. Truncation Strategy and Structural Analysis of an α-Amanitin Aptamer

**Figure 10:**
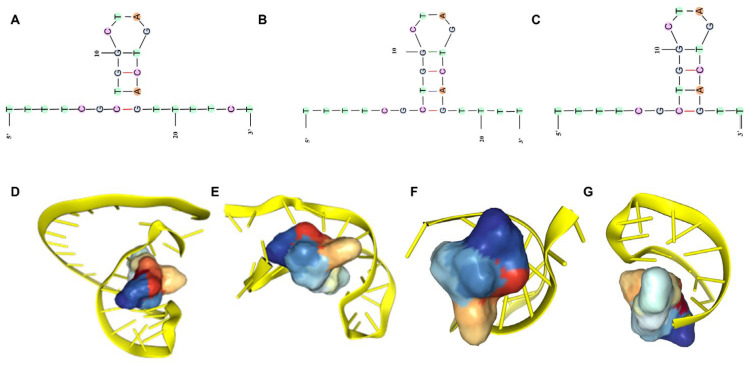
(**A**–**C**) Secondary structures of the truncated aptamers 78-4_trunc1, 78-4_trunc5, and 78-4_trunc6. (**D**–**G**) Molecular docking simulations of the aptamers 78-4, 78-4_trunc1, 78-4_trunc5, and 78-4_trunc6 with α-amanitin.

The stem-loop (hairpin) structure of an aptamer is generally considered the primary recognition and binding site for the target molecule. Accordingly, we first truncated the 3′-terminal segment-CGTCGTGTGAT from the original Seq78-4, while preserving the core stem-loop region and six flanking nucleotides on each side. This initial truncated variant was designated 78-4_trunc1. Starting from 78-4_trunc1, we performed two additional 3′-end truncations, removing two nucleotides each time, yielding the sequences TTTTCGCTGGCTAGTCAGTTTT and TTTTCGCTGGCTAGTCAGTT, which were named 78-4_trunc5 and 78-4_trunc6, respectively (Figure 10 and Table S5). Secondary-structure predictions confirmed that both truncated sequences retained an intact stem-loop, with ΔG values comparable to the original sequence, indicating that thermodynamic stability was not compromised by the truncations.

To evaluate the binding potential of these truncated aptamers with α-amanitin, we performed molecular docking simulations. The resulting interaction data were visualized using Discovery Studio Visualizer (Accelrys Inc., San Diego, CA, USA). Figure 5B illustrates the putative interactions between the aptamer and α-amanitin, as predicted by the docking platform. From the modeled α-amanitin–aptamer complex, the docking results suggest that α-amanitin binds to the loop region of the aptamer structure. Based on a comprehensive evaluation of both Docking Scores and Confidence Scores (Table S6), the truncated variants 78-4_trunc1 (24 nt), 78-4_trunc5 (22 nt), and 78-4_trunc6 (20 nt) exhibited docking metrics comparable to, or even slightly better than, the original 78-4 sequence.

### 2.7. Evaluation of Robustness of Aptamers

According to the stability assay reported by Li et al. [36], the robustness of aptamer Seq78-4 in artificial serum was first investigated, as shown in Figure S8. Compared with the untreated group, Seq78-4 remained structurally intact after 2 h of co-incubation with serum, suggesting that it could maintain stability in serum for at least 2 h without obvious degradation. Meanwhile, as presented in Figure S8, no obvious difference was observed in the electrophoresis bands at 37 °C in comparison with those at 27 °C. These results indicated that the aptamer exhibited favorable stability in the artificial serum under the conditions investigated.

### 2.8. Detection of α-Amanitin in Artificial Serum

The components of the test sample other than the analyte are called matrices, which often interfere with the accuracy of detection results. The artificial serum in the study contained electrolytes, glucose, and human proteins, including human serum albumin and human transferrin, at physiological concentrations. To assess matrix effects, artificial serum was spiked, and dose–response curves were established. Generally, the closer the standard curve of α-amanitin in the actual sample is to that of the buffer sample, the smaller the matrix effect it produces and the higher the recovery [37]. As shown in Figure S7, the dose–response curve under buffer conditions exhibited a limit of detection (LOD) of 1.0 nM and a dynamic range of 1–125 nM. The LOD was determined to be 1.8 nM. In the spiked samples, 10 nM α-amanitin could be clearly detected, while 1 nM could not be distinguished from the blank control, which is consistent with the determined LOD value. Therefore, the method still maintains satisfactory detection sensitivity in the 25-fold diluted solution, enabling reliable quantitation of α-amanitin.

To verify the accuracy of aptamer Seq78-4 for α-amanitin detection in artificial serum, a standard addition method was used with three spiked concentration levels. As shown in Table 2, the recoveries of α-amanitin of all spiked concentrations ranged from 82.2% to 94.1%, with RSDs less than 8.1%. Hence, aptamer Seq78-4 is shown to hold promising potential for the detection of α-amanitin in artificial serum samples. A broader panel of amatoxins and serum proteins will be tested for cross-reactivity in future studies, further supporting the reliability and practical value of the selected aptamer.

## 3. Conclusions

In this study, we successfully utilized a combined approach of target-immobilized SELEX and library-immobilized SELEX to select high-affinity and specific aptamers for the small-molecule α-amanitin. Tosyl-activated magnetic beads and streptavidin beads were employed as separation matrices to perform target-immobilized and library-immobilized selection of the aptamer. Through stringent control of selection conditions, ssDNA sequences with strong binding affinity to α-amanitin were progressively retained. The enrichment of ssDNA sequences increased with each selection round, and the combination of both methods significantly enhanced the selection efficiency. In the target-immobilized SELEX, following fourteen selection rounds with α-amanitin as the target, eleven candidate sequences with binding activity were preliminarily identified. Among them, the representative candidate sequences, designated as Seq14-1 and Seq14-2, exhibited binding affinity to α-amanitin with K_d_ values of 1.88 mM and 1.85 mM, respectively. Subsequently, nine candidate sequences were selected after five rounds of selection in the library-immobilized SELEX. The secondary structures of these nine sequences were classified into three families based on their structural similarities. From each family, representative sequences with lower ΔG values were selected as candidate sequences, leading to the identification of four novel aptamers. Among these, Aptamer Seq78-4 exhibited the highest affinity, with a dissociation constant (K_d_) in the nanomolar range of 57.80 ± 1.001 nM. Furthermore, in terms of counter-selection, aptamer Seq78-4 exhibited significantly higher specificity toward α-amanitin than toward HSA, thus reducing non-specific binding associated with HSA. This work will offer insights for developing aptamer-based sensors for α-amanitin detection and offer a novel strategy for the selection of aptamers against small-molecule targets.

## 4. Materials and Methods

### 4.1. Chemicals and Reagents

All the chemicals and reagents were detailed in the Supplementary Materials.

### 4.2. Selection of the α-Amanitin Aptamer

The target-immobilized SELEX was first used for preliminary screening to enrich aptamer candidates that can specifically bind to α-amanitin and retain maximal abundance of enriched sequences. Firstly, 165 μL of tosyl-activated magnetic beads (loading > 20 μg/mg) were washed and placed in a centrifuge tube, followed by the addition of 125 μL of 700 nM α-amanitin. The mixture was then incubated in coupling buffer at 37 °C for 16–24 h to form an α-amanitin bead solution. Second, a blank control group of α-amanitin incubated with coupling buffer only and a conditional control group of human serum protein (HSA)-modified tosyl-activated magnetic beads were set up under the same conditions. After incubation, PBS with bovine serum albumin (BSA) was added to each group for blocking.

The 500 μL 800 nM library (library sequence: 5′-GGGGAGCTCAGAATAAACGCAA-35N-TTCGACATGAG-3′GCCCGGATC) was incubated with 400 μL α-amanitin-bead solution in a DNA LoBind tube (Eppendorf AG, Hamburg, Germany)at 25 °C for 60 min. After incubation, the tube was placed on a magnetic separator, the supernatant was subsequently discarded, and the precipitate was collected for PCR amplification. We prepared a 50 μL PCR reaction mixture containing 2.5 μL each of the forward primer (GGGAGCTCAGAATAAACGCTCAA) and reverse primer (biotin-GATCCGGGGCCTCATGTCGAA) working stocks, 25 μL of 2× Taq Master Mix buffer, 15 μL of nuclease-free water, and 5 μL of template.

The PCR conditions were as follows: reaction cycles 20 times, 95 °C pre-denaturation for 3 min, 94 °C denaturation for 30 s, 54–61 °C annealing for 1 min, 72 °C extension for 1 min 30 s, 72 °C final extension 5 min, 4 °C hold. Meanwhile, a no-template control group was set up, in which the sample template DNA was replaced with ultrapure water. A total of 500 μL PCR-amplified double-stranded product was added to streptavidin–agarose beads washed by PBS, and the sample was then incubated for 1 h at room temperature on a rotating mixer. After centrifugation and washing, 500 μL of 200 mM NaOH was added to the precipitate, and the mixture was incubated at room temperature for 10 min. Then the sample was centrifuged at 5500 rpm for 3 min, and the free single-stranded DNA in the supernatant was collected. The single-stranded DNA was purified using a desalting column (GE Healthcare illustra NAP-5 column Sephadex G-25 DNA grade) as the secondary library for the next round of screening.

Counter selection: tosyl-activated magnetic beads are modified with reactive tosyl groups on their surface, which can undergo nucleophilic substitution reactions with the amino groups on the surface of the HSA [37], forming stable covalent bonds and thereby enabling the immobilization of HSA on the magnetic bead surface, as shown in Figure S9. Therefore, to eliminate aptamer sequences that non-specifically bind to HSA, after the 4th round of screening, the conditional control group of HSA-modified tosyl-activated magnetic beads was used for counter-selection every 2 rounds. Specifically, HSA-modified tosyl-activated magnetic beads were incubated with the secondary library at 25 °C for 60 min. Sequences with non-specific binding to HSA were captured by the magnetic beads, while α-amanitin-specific aptamers remained in the supernatant. The supernatant was then subjected to the same amplification–enrichment–purification steps as the secondary library. Then the supernatant was taken to the same amplification–enrichment–purification steps as the secondary library. These rounds were repeated 14 times to discover nucleic acid aptamers with high affinity.

However, the target-immobilized SELEX may suffer from hindrance caused by target immobilization on beads, which reduces the aptamers’ affinity. It should be noted that conventional single-target-immobilized SELEX has inherent limitations: target-immobilized SELEX is prone to steric hindrance that may alter the natural conformation of α-amanitin and block its binding sites, thereby reducing the affinity of the screened aptamers; while library-immobilized SELEX alone is likely to cause non-specific binding between the DNA library and magnetic beads, leading to false positive enrichment. Therefore, we continued to employ the library-immobilized SELEX for further selection. This approach avoids alterations to the target’s natural structure or blockage of its binding sites caused by chemical modification, which in turn effectively improves the specificity and affinity of the candidate aptamers by ensuring that the target maintains its native conformation during the screening process.

For library-immobilized SELEX, a new combinatorial library was created by combining a new random library sequence (5′-GGGGAGCTCAGAATAAACGCAA-35N-TTCGACATGAG-3′GCCCGGATC) with a library initially screened from target-immobilized SELEX. A total of 68 μL of 10 μM biotin-labeled capture strand (5′-TTGAGCGTTTATTCTGAGCTCCC-biotin) was hybridized with 500 μL of the combinatorial library. Then, the combinatorial library was coupled to streptavidin-coated magnetic beads through a biotin–avidin interaction, by incubating 400 µL cleaned streptavidin-modified magnetic beads and 500 µL hybridized strands at 25 °C for 1 h with gentle shaking. Then, the library beads were washed to remove unfixed library complementary strands. At each wash, the library beads were transferred to a new DNA Lo-Bind tube. After cleaning, the library beads were obtained. Subsequently, 200 μL of 1× TES buffer was added to the library beads, and the mixture was incubated at 25 °C for 60 min. The supernatant was discarded by magnetic separation to remove released strands without the presence of the target. The target α-amanitin and the library beads were incubated at room temperature for 60 min, after which the supernatant was collected as the library target. A sum of 10 μL of the library target was used as a template for PCR amplification (the PCR amplification and cycling conditions were the same as the target-immobilized SELEX). Next, the PCR products were immobilized on the cleaned streptavidin–agarose beads and subjected to alkaline treatment to prepare ssDNA. After desalting, secondary libraries were obtained (the experimental method was the same as the target-immobilized SELEX). Finally, α-amanitin aptamers with high affinity were obtained after multiple rounds of screening.

This combined strategy combines the advantages of both approaches: the target-immobilized SELEX ensures the preliminary enrichment of α-amanitin-specific aptamer candidates with high abundance, while the library-immobilized SELEX makes up for the defects of target immobilization and improves the affinity and specificity of the candidates. It effectively overcomes the drawbacks of single SELEX and ensures the screening of high-affinity α-amanitin-specific aptamers.

### 4.3. Sequencing and Structure Prediction of the Sequences

The single-stranded DNA (ssDNA) obtained from nucleic acid aptamer screening was then PCR amplified, and high-throughput sequencing analysis was performed of the ssDNA sequences using the Illumina HiSeq 2000 sequencer platform (Illumina, Inc., San Diego, CA, USA) to determine the enriched nucleic acid aptamer sequences. As a result of sequencing, FastQ files were generated using QIIME2 v2019.7, the quality was checked with FastQ, and a reliability analysis of the sequencing results was carried out. The top 20 sequences with the highest degree of homology were selected using MEGA X software. Repeated sequences were classified according to their similarity; a black shaded background indicates 100% homology, whereas gray shaded backgrounds indicate > 50% homology.

Excessively long aptamer sequences with significant spatial site resistance readily impact the binding of the aptamer to the target. Moreover, an overly long aptamer is not beneficial for design and practical applications, as it raises the synthesis cost. Therefore, in this study, the secondary structure of the α-amanitin aptamer was simulated using mFold software. The secondary structure of both random sequence and fixed sequence was simulated, which were classified according to the similarity of secondary structure. Homology analysis and secondary structure prediction were employed for the selection of sequences, and five sequences, 14-1, 14-14, 14-2, 14-3, and 14-6, were finally selected for further investigation.

### 4.4. Aptamers Affinity Test

The affinity of the nucleic acid aptamers was expressed in the dissociation constant (K_d_), which was calculated using the Langmuir isothermal binding model through the equation:

$$K_{d}=\frac{\left[A\right]\cdot [T]}{\left[B\right]}$$

A is the concentration of nucleic acid, T is the concentration of α-amanitin, and B represents the concentration of the library target; free α-amanitin binds the aptamers, causing the aptamers to dissociate from the immobilized beads and remain in the supernatant, whose concentration is then determined for subsequent analysis [32]. All affinity measurements were performed in three independent experiments. Non-linearity was evaluated by calculation of the regression equations through the method of least squares for each curve. Error bars represented as mean ± standard deviation (SD) to reflect experimental reproducibility.

Before the experiment, the concentrations of the candidate aptamers and α-amanitin were determined using a Nanodrop 2000 spectrophotometer (Thermo Fisher Scientific, Waltham, MA, USA) with the Nucleic Acid mode and Protein mode, respectively. A total of 100 μL of 10 μM candidate aptamers was incubated with 206 μL of washed streptavidin-coated magnetic beads conjugated with the capture strand to obtain the library beads. Then, 150 μL of 20 μM α-amanitin was mixed thoroughly with the washed library beads, and the mixture was incubated on a rotating shaker at 25 °C for 60 min. After incubation, the tube was placed on a magnetic stand for 2 min, and the supernatant was collected for the determination of the library-target concentration. The concentration of the library target was determined using the Nucleic Acid mode. All of the experiments were performed in triplicate to ensure data accuracy, and the average of the triplicate data was used for the analysis.

### 4.5. Aptamers Specificity Test

In order to test the specificity of aptamer Seq78-4 for α-amanitin, three groups were set up: target α-amanitin group A, control group B (the target human serum protein (HSA)), and blank control group C (the target 1× binding buffer). The concentrations of the aptamer before their incubation with A, B, and C groups on the magnetic beads were recorded as A1, B1, and C1, respectively, and the concentrations of the aptamer after incubation with the target were recorded as A2, B2, and C2, respectively. The concentration differences among the three groups (the differences between A1 and A2, B1 and B2, and C1 and C2) were compared.

Firstly, the library was coupled to streptavidin-modified magnetic beads through biotin–avidin interaction. Next, 200 μL 1× TES buffers were incubated with library beads at 25 °C for 60 min. After magnetic separation, 200 μL supernatant was retained for the nucleic acid concentration measurement. Finally, the targets α-amanitin, HSA, and 1× binding buffer were separately incubated with library beads for 60 min at room temperature. The supernatant was taken for measurement after magnetic separation. To ensure data accuracy, each datum was measured three times to obtain the average value.

### 4.6. Serum and Temperature Stability of Seq784

The aptamer Seq-78-4 was incubated in a 25-fold diluted artificial serum to evaluate its serum stability and temperature stability, respectively. For serum stability assessment, the aptamer was incubated at room temperature for 0, 30, 60, and 120 min. For temperature stability assessment, it was incubated at 27 and 37 °C for 30 min, respectively. All samples were separated by 3% agarose gel electrophoresis, and the degradation of the aptamer was observed using a gel imaging system.

### 4.7. Sample Preparation and Extraction

A series of different concentrations (0 nM, 1 nM,10 nM, 25 nM, 50 nM, 75 nM, 100 nM, and 125 nM) of α-amanitin was applied onto the surface of tosyl-activated magnetic beads to form α-amanitin beads, and then incubated with an equal amount of Seq78-4 in TES buffer and artificial serum. After magnetic separation, the supernatant was collected to detect the Seq78-4 concentration. As the concentration of α-amanitin in the system decreased, the amount of Seq78-4 bound to α-amanitin decreased, and the concentration of unbound Seq78-4 in the supernatant increased accordingly. The binding rate was calculated as follows:

Binding (%) = (A0 − A)/A0 × 100

A0 represents the initial concentration of aptamer Seq78-4, and A denotes the concentration of aptamer Seq78-4 in the supernatant after incubation. The minimum detection limit is defined as the concentration of analyte that inhibits the binding observed at zero analyte by a significant level, in excess of 3SD values.

Accuracy was assessed by determining the recovery of samples (blank artificial serum) spiked with three levels of standard α-amanitin solutions. Precision was assessed by the relative standard deviation (RSD) of the recovery of three replicates.

## Figures and Tables

**Table 1:** The selection conditions in each target-immobilized SELEX round.

Rounds	Ratio of Library to Target	Incubation Time (min)	Elution Times	Counter Selection
1	10:1	30	3	
2	7.5:1	30	3	
3	7:1	30	6	
4	6:1	30	3	√
5	6:1	30	6	
6	5:1	30	3	√
7	5:1	30	3	
8	5:0.66	30	3	√
9	4:0.66	30	6	
10	3.5:0.66	30	6	√
11	3.5:0.66	20	6	
12	3.5:0.66	20	6	√
13	3.5:0.66	20	6	
14	3.5:0.66	20	6	√

√ indicates that counter selection was performed in the corresponding SELEX round.

**Table 2:** Recovery of α-amanitin from spiked artificial serum (*n* = 6).

Compounds	Added (nM)	Recovery (%)	RSD (%)
α-Amanitin	25	82.2	8.1
	50	87.2	7.7
	100	94.1	6.3

## Data Availability

The original contributions presented in this study are included in the article/Supplementary Materials. Further inquiries can be directed to the corresponding author.

## Supplementary Materials

The following supporting information can be downloaded at: https://www.mdpi.com/article/10.3390/toxins18040163/s1. Figure S1. Chemical structures of the α-amanitin variants examined in this paper. Figure S2. Seq14-2 base mass distribution map. The X axis is the position of the base in reads, and the Y axis is the base quality value. Green part in the figure represents High-quality bases (Phred score ≥ 30), yellow part in the figure represents good quality bases (20 ≤ Phred score < 30), pink part in the figure represents poor quality bases (Phred score < 20). Table S1. Results of secondary structure simulation analysis for sequences without fixed regions. Table S2. Results of Secondary structure simulation analysis complete sequences with fixed sequence. Figure S3. Concentration of unbound ssDNA in the library-immobilized SELEX rounds after the 1st to 4th washes with TES buffer. W1, W2, W3, and W4 represent the 1st, 2nd, 3rd, and 4th washes, respectively. Figure S4. Optimization of PCR cycles in the library-immobilized SELEX. Table S3. Selected oligonucleotide sequence candidates. Nine most frequent oligonucleotide sequences obtained after HTS of the 5th Library-immobilized SELEX. Table S4. Comparison of the aptamers affinity obtained by Target-immobilized and Library-immobilized SELEX with other aptamers known to bind α-amanitin. Figure S5. Three families of predicted partial secondary structures of the 9 most frequently repeated aptamers in the library-immobilized SELEX. Figure S6. Determining α amanitin aptamers affinity by gold nanoparticle (AuNP)-based method. Table S5. Primitive and truncated aptamer sequences (5’-3’), melting Temperature (Tm), and dG values. Table S6. Molecular docking metrics of α-Amanitin with aptamer (78-4, 78-4_truncl, 78-4_trunc5 and 78-4_trunc6). Figure S7. Evaluation of placement stabilities of Seq78-4 in artificial serum. Figure S8. α-Amanitin dose–response graphs carried out as described where the standards were made up in buffer and artificial serum. Figure S9. Schematic of α-amanitin aptamer selection via counter selection. S2.1 Chemicals and reagents. Table S7. Sequences required in target-immobilized SELEX and library-immobilized SELEX. S2.2 Aptamer Affinity Tests based on the AuNPs S2.3 Truncation of the α-amanitin Aptamer Sequence
