# Supplementary material for: Combined Target-Immobilized and Library-Immobilized SELEX for Selecting High-Affinity α-Amanitin Aptamers
Source: Toxins (Basel). 2026 Mar 30;18(4):163. doi: 10.3390/toxins18040163 (PMC13120037; doi:10.3390/toxins18040163)
Supplement: Supplementary file 1 [file toxins-18-00163-s001.zip › toxins-4147541-supplementary.pdf]

Supplementary Materials

Combined Target-Immobilized and Library-Immobilized SELEX for Selecting High-Affinity α-Amanitin Aptamers

Yang Li [1,†], Muling Shi [1,2,*,†], Wenyue Li [1], Yiqing Yang [3], Xiang Li [3], Chen Shen [3], Xiong Wang [3], Shuanglin Zhang [3] and Jie Du [1]

[1] State Key Laboratory of Tropic Ocean Engineering Materials and Materials Evaluation, School of Materials Science and Engineering, Hainan University, Haikou 570228, China; 23210805000002@hainanu.edu.cn (Y.L.); wenyueli@hainanu.edu.cn (W.L.); dujie@hainanu.edu.cn (J.D.)

[2] Molecular Science and Biomedicine Laboratory, College of Biology, Hunan University, Changsha 410082, China

[3] Hunan Provincial Key Laboratory of Forestry Biotechnology, College of Life Science and Technology, Central South University of Forestry & Technology, Changsha 410004, China; yyq970504@163.com (Y.Y.); lx534976886@gmail.com (X.L.); charybdis233@gmail.com (C.S.); wangxiongvincent@163.com (X.W.); zslzlt@163.com (S.Z.)

* Correspondence: mulingshi@hnu.edu.cn

† These authors contributed equally to this work.

S1. Supplementary Table and Figure

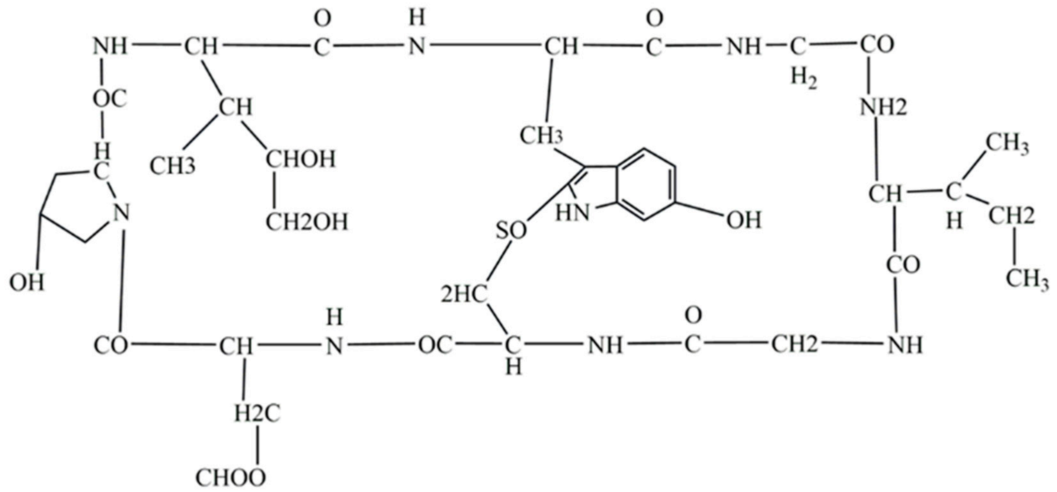

Figure S1. Chemical structures of the α-amanitin variants examined in this paper.

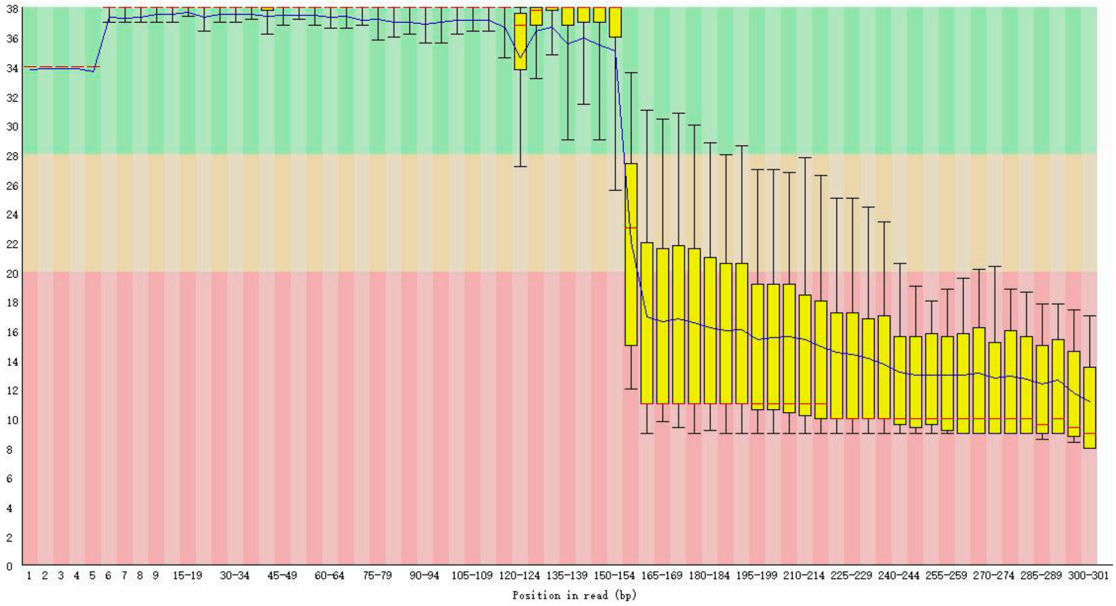

Figure S2. Seq14-2 base mass distribution map. The X axis is the position of the base in reads, and the Y axis is the base quality value. Green part in the figure represents High-quality bases (Phred score >=30), yellow part in the figure represents good quality bases (20<=Phred score <30), pink part in the figure represents poor quality bases (Phred score <20).

Table S1. Results of secondary structure simulation analysis for sequences without fixed regions.

Sequence	Secondary Structure	Sequence	Secondary Structure
	Structure Template One		
8-1	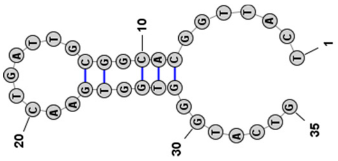	14-1	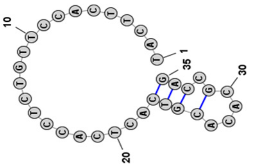
8-7	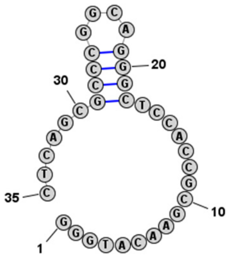	14-3	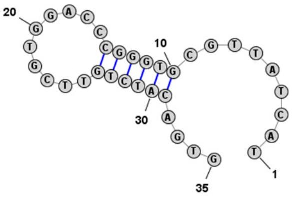
14-10	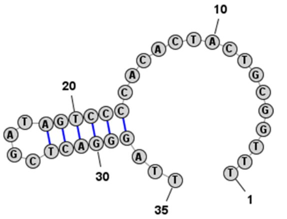		
	Structure Template Two		
8-4	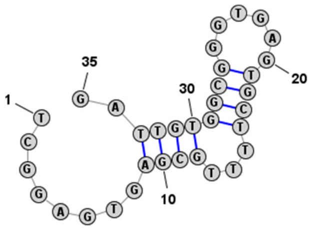	14-2	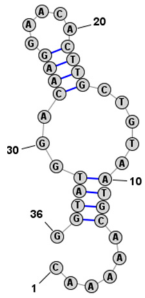
	Structure Template Three		

Sequence	Secondary Structure	Sequence	Secondary Structure
14-4	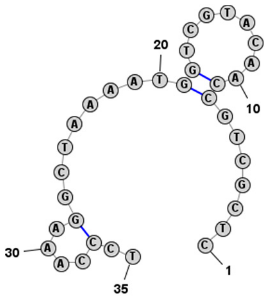	14-16	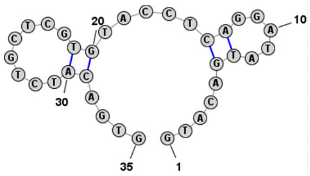
	Structure Template Four		
14-5	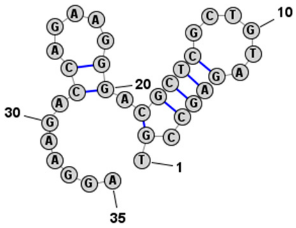	14-15	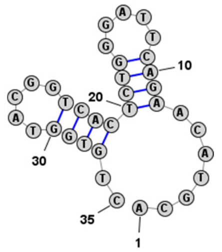
	Structure Template Five		
14-14	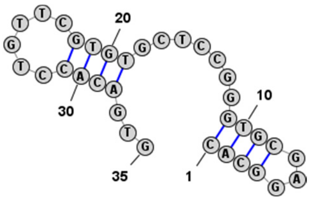	14-17	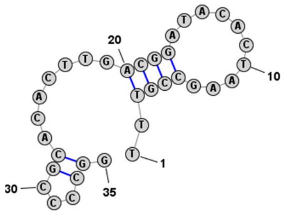
	Structure Template Six		
14-6	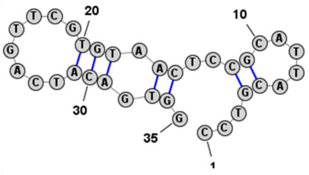		
	Structure Template Seven		

Sequence	Secondary Structure	Sequence	Secondary Structure
14-7		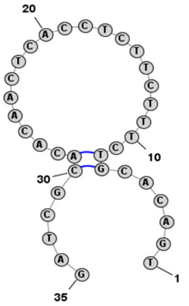	
Structure Template Eight			
14-19	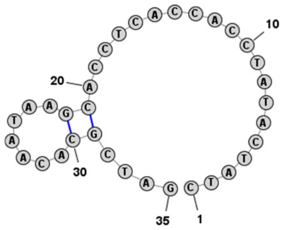		

Table S2. Results of Secondary structure simulation analysis complete sequences with fixed sequence.

**Sequence**	**Secondary Structure**	**Sequence**	**Secondary Structure**
	Structure Template One		
8-1	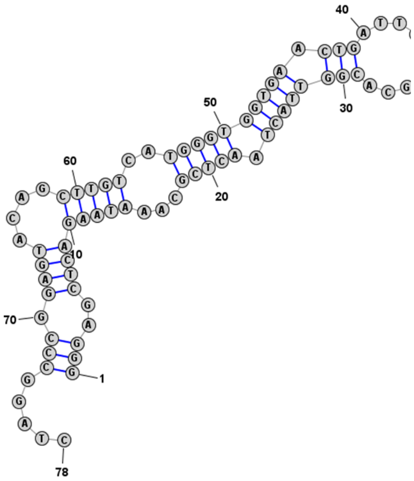	14-5	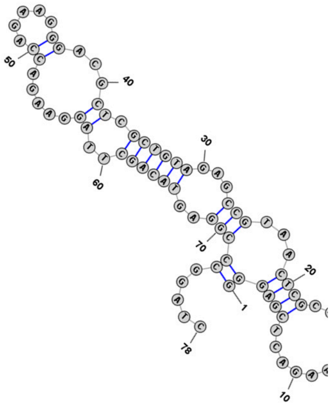
	Structure Template Two		
8-4	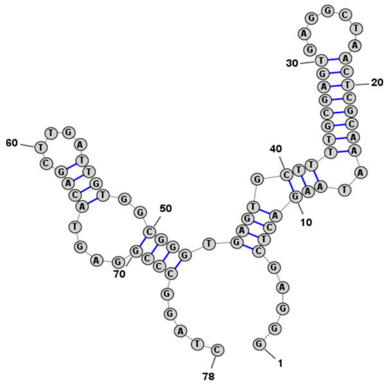	14-14	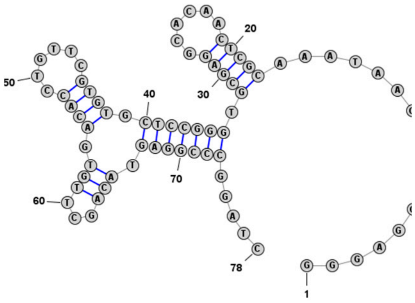
	Structure Template Three		
14-1	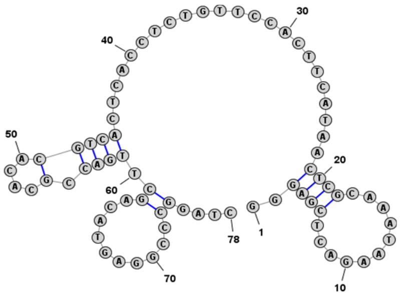	14-17	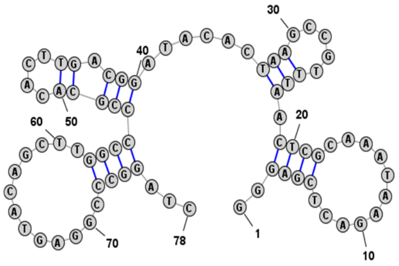

Sequence	Secondary Structure	Sequence	Secondary Structure
	Structure Template Four		
14-2	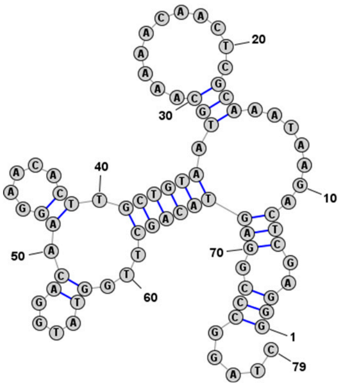	14-10	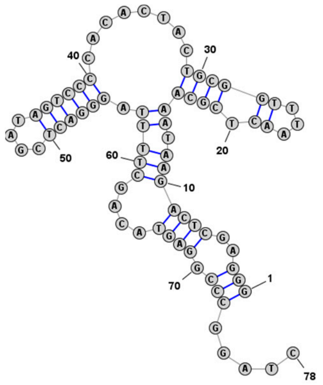
	Structure Template Five		
14-3	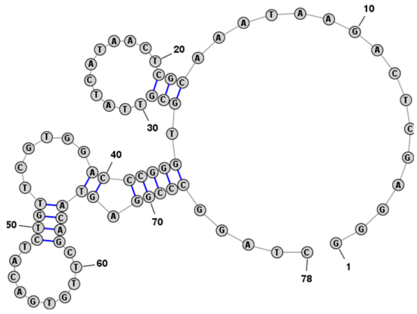	14-7	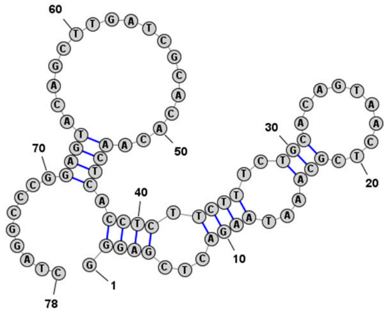
	Structure Template Six		

Sequence	Secondary Structure	Sequence	Secondary Structure
14-4	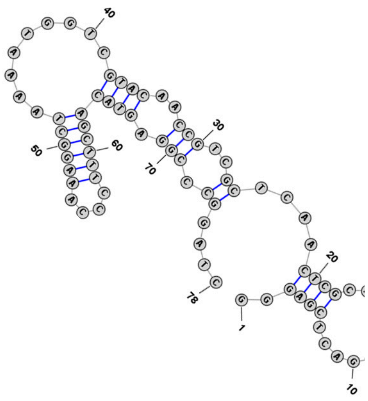	14-19	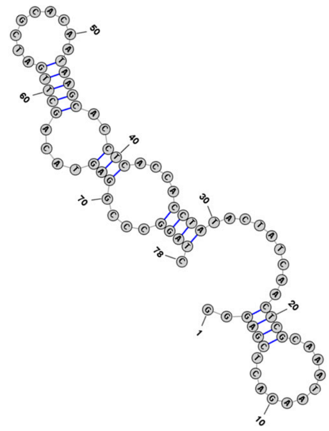
Structure Template Seven			
14-6	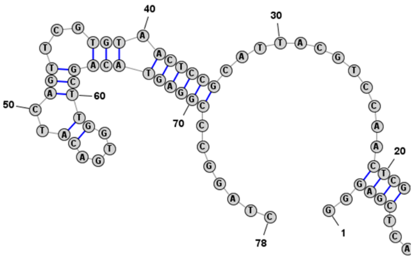	14-15	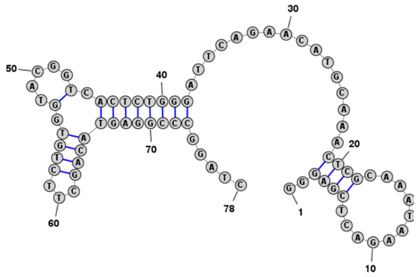
14-16	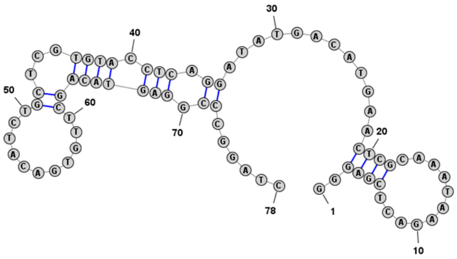		
Structure Template Eight			
8-7	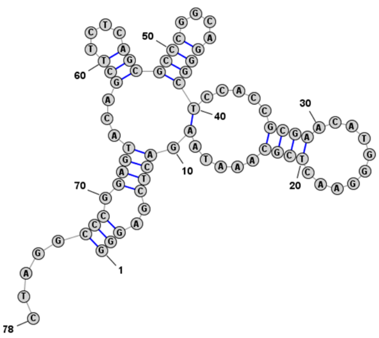		

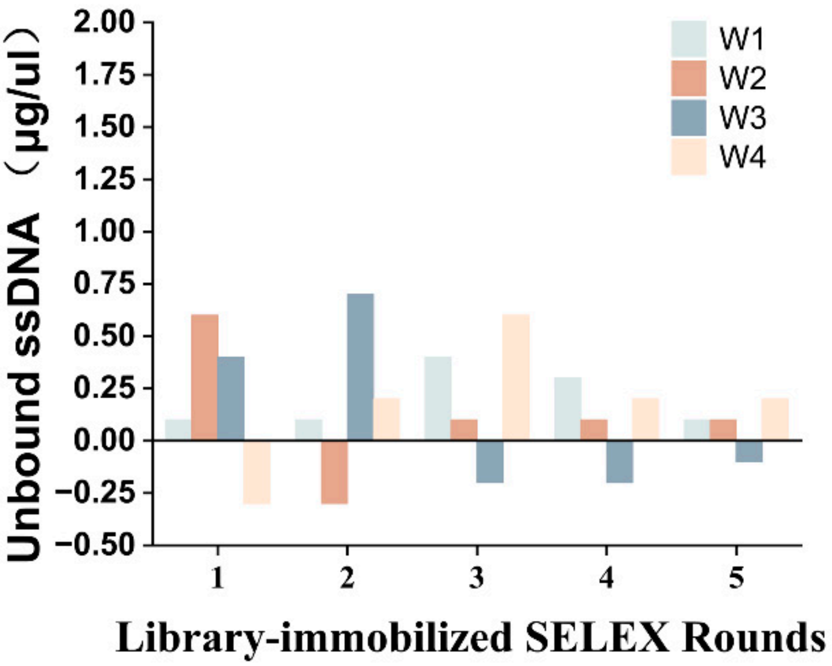

Figure S3. Concentration of unbound ssDNA in the library-immobilized SELEX rounds after the 1st to 4th washes with TES buffer. W1, W2, W3, and W4 represent the 1st, 2nd, 3rd, and 4th washes, respectively.

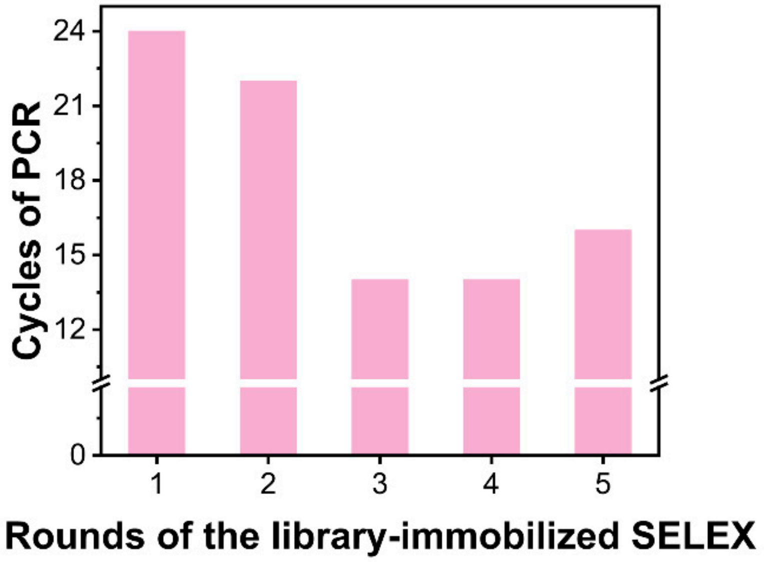

Figure S4. Optimization of PCR cycles in the library-immobilized SELEX.

Table S3 Selected oligonucleotide sequence candidates. Nine most frequent oligonucleotide sequences obtained after HTS of the 5th Library-immobilized SELEX.

Aptamer	Sequence（5'-3’）	Copies
Seq78-1	TTGTTCTTTAGTCCTAATTTGCAGGTTTAATCTTC	426
Seq78-2	GCACGCGTCGTCTTCCTGCGCTTCTCCCCGCATCA	314
Seq78-3	TGTAGACTTTTTACCTGGTTCTTAACATTTTAATG	237
Seq78-4	TTTTCGCTGGCTAGTCAGTTTTCTCGTCGTGTGAT	196
Seq78-5	TTGATGATCTCTTCTCCTTTTTCTTTTTAGGCTAG	134
Seq78-6	TATTACTTCCGGTGTCGTAGTCAGTTTTTTAGTTA	98
Seq78-7	GTTCCTCTATTCTAGTTATGCGCTACGTACGCTAG	75
Seq78-8	TGGGCTACATTTAGCGCTTTATATTTCACGCCACG	42
Seq78-9	CTTTTCTGGTTACTCTCGTTTGTTTGATGTTACTT	28

Table S4 Comparison of the aptamers' affinity obtained by Target-immobilized and Library-immobilized SELEX with other aptamers known to bind α-amanitin.

No.	Aptamer	Kd	Reference
1	Seq78-2	0.85 μM	Our work
2	Seq78-4	57.8 nM	
3	Seq78-7	256.6 nM	
4	Seq78-9	0.91 μM	
5	Ama1	5.026 μM	[18]
6	α-30	47.65 nM	[25]
7	α-30-2	42.04 nM	
8	α-30-3	52.85 nM	
9	α-30-1	63.66 nM	
10	α-30-4	91.89 nM	
11	Apt	33.6 nM	[24]
12	Apt-2	37.9 nM	
13	Apt-8	130.9 nM	
14	Apt-10	108.7 nM	
15	Apt-11	184.4 nM	
16	Apt-12	185.3 nM	
17	Apt	3.1 μM	[33]
18	Apt-2	46.9 μM	
19	Apt-8	11.9 μM	
20	Apt-10	68 μM	
21	Apt-11	54.6 μM	
22	Apt-12	84.7 μM	

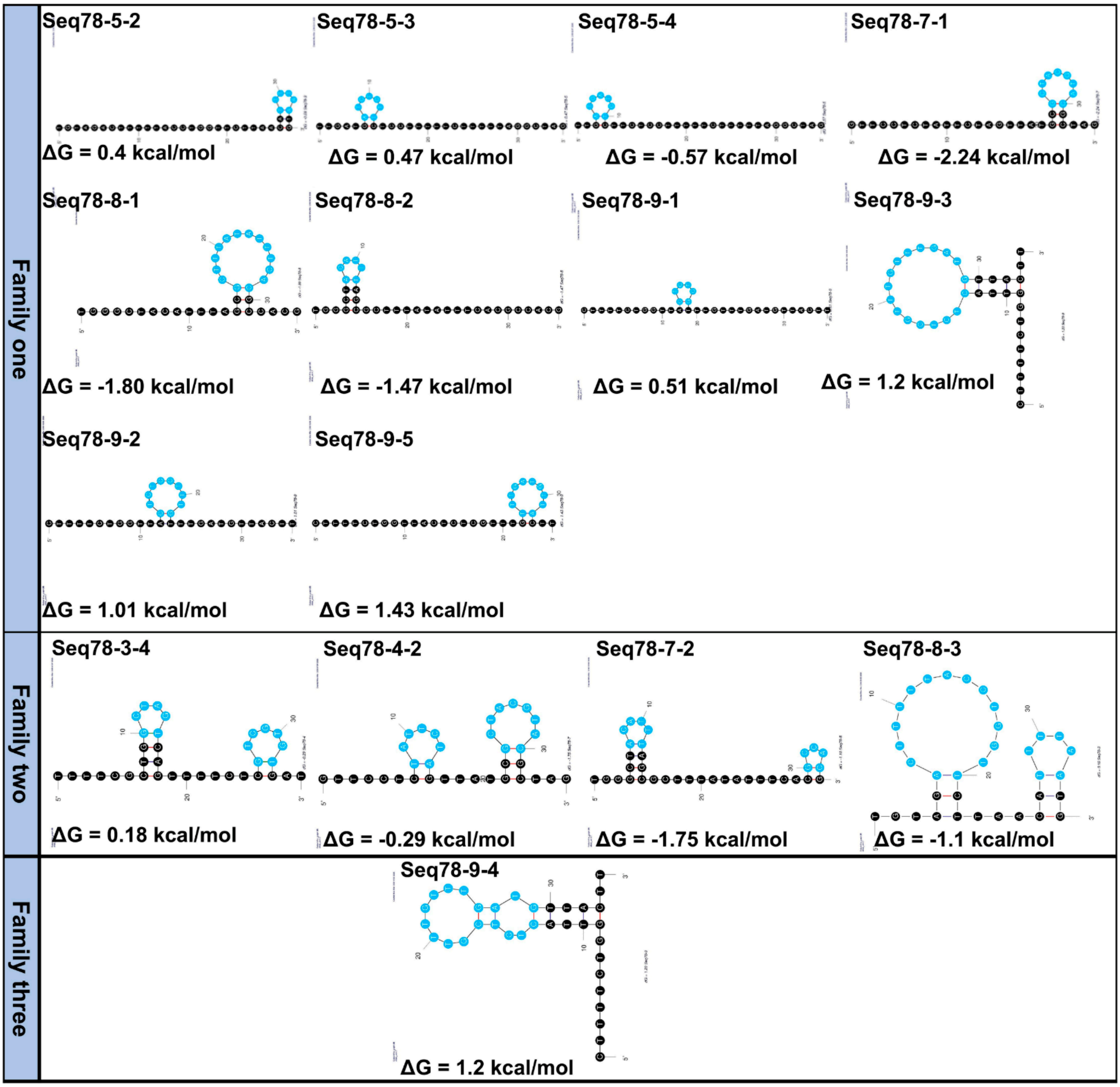

Figure S5. Three families of predicted partial secondary structures of the 9 most frequently repeated aptamers in the library-immobilized SELEX.

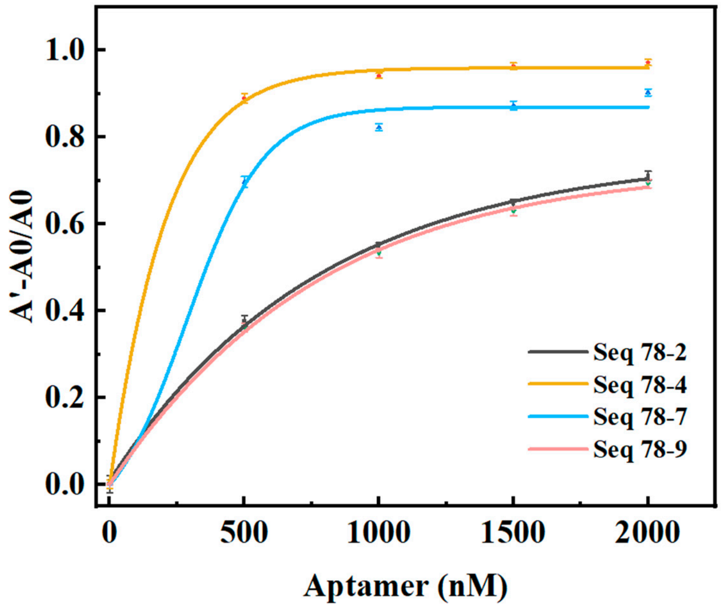

Figure S6 Determining α-amanitin aptamers affinity by gold nanoparticle (AuNP)-based method.

Table S5 Primitive and truncated aptamer sequences (5'-3'), melting Temperature (Tm), and dG values.

Name	Sequence (5'-3')	dG (kcal/mol)	Tm (°C)
78-4	TTTTCGCTGGCTAGTCAGTTTTCTCGTCGTGTGAT	-1.12	50.6
78-4_trunc1	TTTTCGCTGGCTAGTCAGTTTTCT	-1.12	50.6
78-4_trunc5	TTTTCGCTGGCTAGTCAGTTTT	-1.12	50.6
78-4_trunc6	TTTTCGCTGGCTAGTCAGTT	-1.12	50.6

Table S6 Molecular docking metrics of α-amanitin with aptamer (78-4, 78-4_trunc1, 78-4_trunc5 and 78-4_trunc6).

**α-Amanitin combines with**	**78-4_**	**78-4_trunc1**	**78-4_trunc5**	**78-4_trunc6**
Docking Score	-262.11	-264.83	-254.26	-241.22
Confidence Score	0.9040	0.9086	0.8895	0.8611
Ligand RMSD (Å)	31.76	20.96	28.11	26.15

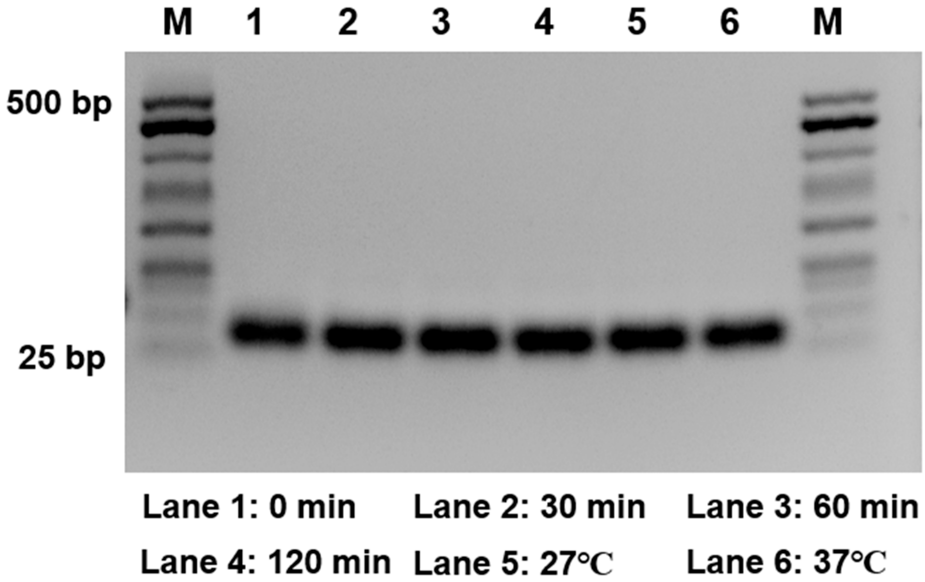

Figure S7. Evaluation of placement stabilities of Seq78-4 in artificial serum.

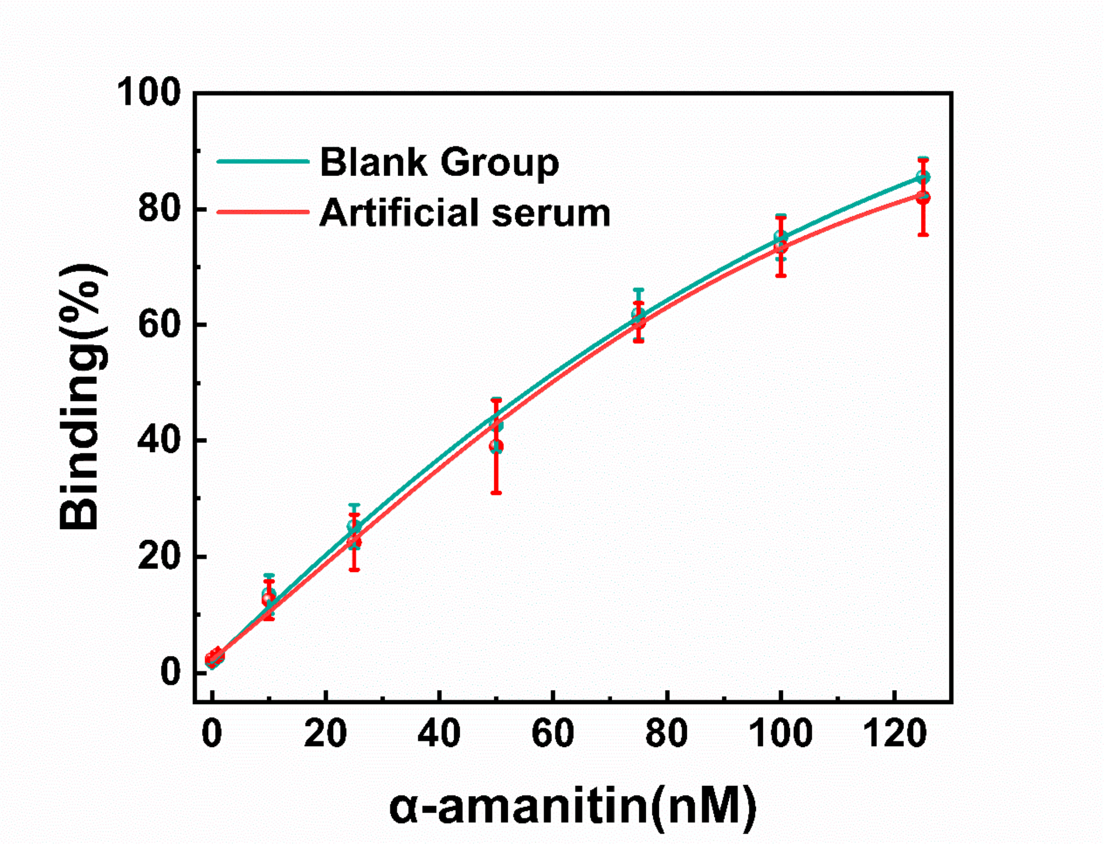

Figure S8. α-Amanitin dose-response graphs carried out as described where the standards were made up in buffer and artificial serum.

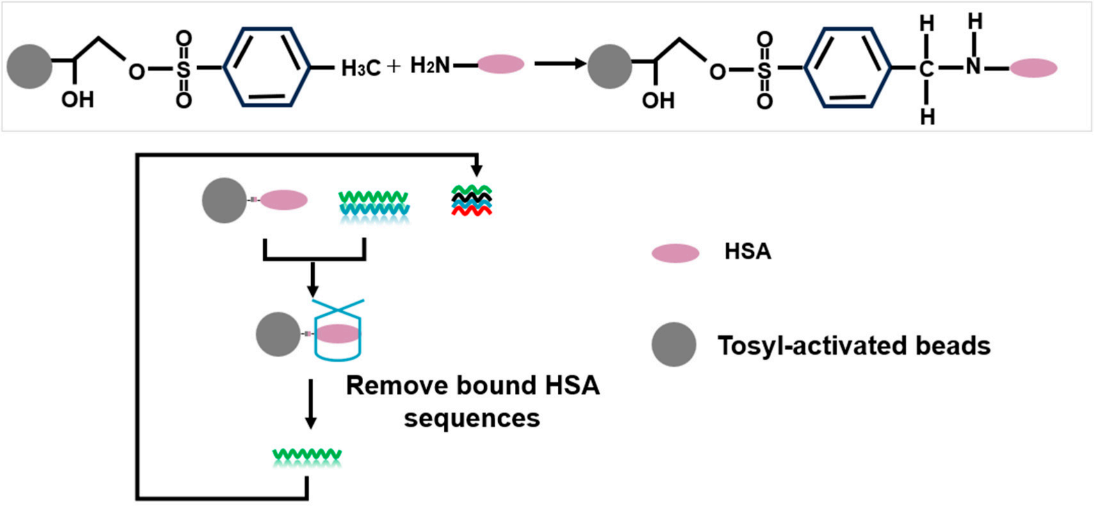

Figure S9 Schematic of α-amanitin aptamer selection via counter selection.

S2. Supplementary Experiments

S2.1 Chemicals and reagents

All of the DNA samples utilized in this study were custom-synthesized and purified by high-performance liquid chromatography (HPLC) from Shanghai Sangong Bioengineering Technical Co., Ltd (Shanghai, China). The forward and reverse primers for polymerase chain reaction (PCR) were purchased from Takara Bio (Dalian, China), which were also purified by HPLC. All the sequences mentioned above are listed in Table S7. Dynabeads M-280 Tosylactivated and Dynabeads M-270 Streptavidin, Invitrogen, 2.8 mum diameter were purchased from Thermo Fisher Scientific (Waltham, MA, USA). DNA marker was purchased from BBI Life Sciences (Shanghai, China). 6×DNA loading buffer was obtained from Takara Bio (Dalian, China). α-Amanitin was purchased from MCE (Shanghai, China). 2×Taq master mix was obtained from Novoprotein (Beijing, China). All chemicals used in this experiment were of analytical grade. Sodium hydroxide (NaOH) was purchased from Tianjin Hengxing Chemical Reagent Co. (Tianjin, China). Sodium chloride (NaCl), sodium dihydrogen phosphate ($NaH_2PO_4 \cdot H_2O$), ammonium sulfate $(NH_4)_2SO_4$), and boric acid (H_3BO_3) were purchased from Shanghai Hushi Chemical Reagent Co. (Shanghai, China). Agarose gel was obtained from Juhemei (Beijing, China); Tris(hydroxymethyl)aminomethane Tris-HCl (2 M, 500 mL, pH 7.5) was bought from Sangon Biotech Co. (Shanghai, China);. Bovine serum albumin (BSA, purity > 98%) and GelRed nucleic acid dye were purchased from Beyotime Biotechnology (Shanghai) Co. (Shanghai, China). AuNPs were purchased from BBI Solutions (Crumlin, UK).

Table S7 Sequences required in target-immobilized SELEX and library-immobilized SELEX.

Name	Base sequence（5' to 3'）
F-am	GGGAGCTCAGAATAAACGCTCAA
R-am	GATCCGGGCCTCATGTCGAA
R-am-biotin	biotin-GATCCGGGCCTCATGTCGAA
lib78-am	GGGAGCTCAGAATAAACGCTCAANNNNNNNNNNNNNNNN NNNNNNNNNNNNNNNNNNNNTTCGACATGAGGCCCGGATC
Capture-am	TTGAGCGTTTATTCTGAGCTCCC
Capture-am-biotin	TTGAGCGTTTATTCTGAGCTCCC-biotin
8-1	TCATTGGCACGGCGTTAGTCAAGTGGTGGGTACTG
14-1	TACTTCACCTTGTCTCCACTCACTGCACACGCCAG
14-2	CAAAAACGTAATGTCGTTCACAAGGAACAGGTATGG
14-4	CTCGCTGCCAACATGCTGGTAAAATCGGAAACCCT
14-5	TGCCGAGATGTCGCTCGCAGGGAAGACCAGAAGGA
14-6	CCTGCATTACGCCTCAATGTGCTTGACTACAGTGG
14-14	CACGGAGCGTGGGCCTCGTGTGCTTGTCCACAGTG

14-17	TTTGCCGAATCACATAGGCAGTTCACACGCCCCGG
14-19	CTATCATATCCACCACTCCACGAATAACACGCTAG
8-7	AGTTTGTGTGAAACTACTTCGGATGGCTTAACCCT
14-3	TACTATTGCGTGGGCCCAGGTGCTTGTCTACAGTG

S 2.2 Aptamer Affinity Tests based on the AuNPs

Different concentrations of the aptamer (0, 50, 100, 200, 400, 800, and 1600 nM) were incubated with 1 ug/mL of α-amanitin for 30 min at room temperature. AuNPs were added and incubated for 30 min at room temperature, followed by the addition of 1 M NaCl, to make a final concentration of 40 mM. Absorbance values at 520 nm were measured using a Nanodrop 2000 spectrophotometer (Thermo Fisher Scientific, Waltham, MA, USA). The aptamer concentration was used as the horizontal coordinate and (A' – A0)/A0 was the vertical coordinate, and the aptamer affinity constants were calculated usingGraphPad Prism software (Version 8.0, GraphPad Software, Inc. La Jolla, CA, USA).A' represented the A520 nm value of the aptamer at each concentration. A0 represented the A520 nm value when the aptamer concentration was zero.

S 2.3 Truncation of the α-amanitin Aptamer Sequence

Based on our previous work, Seq78-4 was identified as an effective α-amanitin aptamer, with the following nucleotide sequence: 5'-TTTTCGCTGGCTAGTCAGTTTTCTCGTCGTGTGAT-3'. We performed secondary structure prediction of 78-4 again using mFold to confirm the accuracy of our earlier findings. From the predicted secondary structure, we identified a key stem-loop motif corresponding to the sequence CTGGCTAGTCAG. Notably, the 3' end appeared somewhat elongated and did not participate in this characteristic stem-loop structure. Therefore, we decided to truncate the 3' terminal segment -CGTCGTGTGAT while preserving the stem-loop region along with 6 nucleotides on each flank. This reduced the sequence length from 35 nt to 24 nt, and this initial truncated variant was designated as 78-4_trunc1. Starting from 78-4_trunc1, we performed two additional truncations from the 3' end, removing two nucleotides each time. This yielded the sequences TTTTCGCTGGCTAGTCAGTTTT and TTTTCGCTGGCTAGTCAGTT, designated as 78-4_trunc5 and 78-4_trunc6, respectively, representing an attempt to minimize DNA chain length. The three-dimensional structure of α-amanitin was obtained from the Research Collaboratory for Structural Bioinformatics PDB (RCSB PDB) database (http://www.rcsb.org/pdb) as a CIF file, which was subsequently converted to PDB

format using PyMOL (The PyMOL Molecular Graphics System, Version 1.5.0.4, Schrodinger, LLC). For the aptamer sequences, tertiary structures were modeled using the RNA composer online server(https://rnacomposer.cs.put.poznan.pl).based on the dot-bracket notation derived from their secondary structures.
